# A multidisciplinary intervention targeting pragmatic language and maternal flexibility in Egyptian children with oppositional defiant disorder

**DOI:** 10.1186/s40359-026-04022-6

**Published:** 2026-02-10

**Authors:** Hoda G. Mohammed, Hassnaa O. Mohammed

**Affiliations:** 1 Department of Child Psychological Studies, Faculty of Post-graduate Childhood Studies, Ain Shams University, Cairo, Egypt; 2 Medical Studies Department for Children, Faculty of Post-graduate Childhood Studies, Ain Shams University, Cairo, Egypt

**Keywords:** Oppositional defiant disorder, Psychological flexibility, Pragmatic language disorders. pragmatics' assessment

## Abstract

**Background:**

Oppositional Defiant Disorder (ODD) is a multifaceted behavioral condition with a growing theoretical and empirical foundation regarding its etiology, risk factors, comorbidities, and developmental trajectories. This complexity necessitates integrated management strategies that simultaneously address co-occurring domains, such as child pragmatic language deficits and maternal psychological inflexibility, beyond the scope of conventional interventions like standard Parent Management Training (PMT). This study aims to determine the outcome of an intervention program that targeted pragmatic skills in children with ODD and their mothers’ psychological flexibility.

**Methods:**

A sample of 100 children with ODD was recruited from the Child Psychiatry Clinic and assessed for co-occurring pragmatic language disorders using the Egyptian Arabic Pragmatics Language Test (EAPLT). Of these, 60 children exhibiting pragmatic deficits were randomly assigned to either Group I, which received the intervention program, or group II, which did not (30 per Group). Both groups were assessed twice, before and after the application of the intervention program, using EAPLT for children, the Maternal Psychological Flexibility Scale (MPFS) for mothers, and the ODD Scale (parents’ form). A third assessment was conducted as a follow-up after one and a half months to examine the longevity of the outcome.

**Results:**

The participants in Group I, children and their mothers, showed significantly higher total pragmatic scores and maternal psychological flexibility scores than those in Group II. This improvement led to a decrease in the severity of ODD, as indicated by the ODD scale score among participants in Group I. The positive outcome of the intervention program remained evident for up to one and a half months following the completion of the rehabilitation program.

**Conclusions:**

Implementing this intervention program for children who experience comorbidity between language disorders and emotional/behavioral disorders is recommended.

## Background

Oppositional Defiant Disorder (ODD) is one of the most prevalent disruptive behavioral disorders in childhood [1]. According to the Diagnostic and Statistical Manual of Mental Disorders, Fifth Edition (DSM-5) [1], ODD characterized by a persistent pattern of angry/irritable mood, argumentative/defiant behavior, and vindictiveness that significantly impairs social, academic, and family functioning [2]. Epidemiological studies estimate community prevalence rates between 2% and 15%, with markedly higher rates in clinical samples. ODD often emerges before age eight, tends to follow a chronic course, and constitutes a well-established risk factor for the development of conduct disorder and other externalizing conditions [3–5].

The etiology of ODD is widely recognized as multifactorial, arising from a dynamic interplay of genetic, neurobiological, temperamental, familial, and socioenvironmental factors [6, 7]. Twin and adoption studies consistently indicate a heritable component [8]. Neurocognitive research further implicates deficits in emotion regulation, executive functioning, and reward processing, often linked to atypical functioning in prefrontal and limbic brain regions [9]. Concurrently, environmental influences, such as harsh or inconsistent parenting, low parental warmth, exposure to community violence, socio-economic adversity, and peer rejection, significantly contribute to both the onset and persistence of ODD symptoms [10]. Importantly, these risk factors do not operate in isolation; rather, they interact in complex, transactional ways over developmental time, shaping individual trajectories of risk and resilience [6, 11].

Within this biopsychosocial framework, two interrelated domains have emerged as particularly salient yet underintegrated in clinical practice: pragmatic language competence in the child and psychological flexibility in the caregiver [12, 13].

Pragmatic language, the ability to use verbal and nonverbal communication appropriately in social contexts, is foundational for interpreting social cues, regulating emotions, resolving conflicts, and maintaining relationships [14, 15]. Children with ODD frequently exhibit significant pragmatic deficits, including difficulty understanding inferences, initiating or sustaining conversations, expressing emotions, and repairing communication breakdowns [16, 17]. These impairments are not merely secondary features; they actively contribute to behavioral escalation. For instance, a child who cannot verbally negotiate a conflict may resort to defiance, while misinterpretations of social intent can fuel irritability [18]. Research indicates that 71–86% of children with externalizing disorders demonstrate clinically significant pragmatic language difficulties [19, 20]. McKinney and Renk [21] further identified lower verbal intellectual functioning among children with ODD, while Alvarez & Ollendick [22] emphasized that social cognition deficits, particularly errors in interpreting and responding to social stimuli, play a cardinal role in the pathophysiology of ODD. Indeed, Mandy et al. [23] posited that such social cognition deficits form the backbone of pragmatic impairments, with ODD and primary pragmatic disorders sharing a core deficit in understanding implicit social rules.

Concurrently, parenting quality, especially the caregiver’s capacity to respond flexibly to stress, plays a pivotal role in the maintenance or amelioration of ODD symptoms [24]. Maternal psychological flexibility, defined as the ability to remain present, adaptively regulate emotions, and persist in value-driven parenting despite distress, has been robustly linked to reduced child externalizing behaviors [25, 26]. Low flexibility is associated with rigid, punitive, or emotionally reactive parenting, which can exacerbate coercive cycles and undermine the child’s developing self-regulation [27]. This relationship is mediated by the child’s emotional regulation, underscoring the developmental interdependence of maternal and child functioning [28].

Critically, these two domains, child pragmatic competence and maternal psychological flexibility, are bidirectionally linked. A child’s communication deficits increase caregiver stress and reduce opportunities for positive interaction, thereby eroding parental flexibility [29, 30]. Conversely, inflexible parenting limits the scaffolding needed for the child to acquire nuanced social communication skills [31]. This transactional loop suggests that interventions targeting only one domain may yield limited or transient effects.

Despite this theoretical synergy, current evidence-based treatments for ODD, such as Parent Management Training (PMT), primarily focus on behavioral compliance and discipline strategies, with minimal attention to underlying language impairments [32, 33]. Similarly, speech-language interventions rarely incorporate family-level emotional or regulatory components.

Despite growing theoretical and empirical interest in the interplay between child pragmatic language impairments and caregiver psychological processes in disruptive behavior disorders, few intervention studies, particularly in low- and middle-income countries, have concurrently targeted both domains. Recent international work underscores this gap: Pritzker [20], in a systematic review of pragmatic language and emotional-behavioral functioning, concluded that integrated, interprofessional models remain rare, especially outside high-income Western contexts. Similarly, Law et al. [34] demonstrated that pragmatic language mediates over half the association between early social disadvantage and adolescent conduct problems in a UK cohort, highlighting its potential as a modifiable mechanism in diverse settings. To date, however, no published trial has evaluated a dual-focus intervention combining pragmatic language therapy and parental psychological flexibility training in an Arabic-speaking or North African population. This limits the cultural and linguistic generalizability of existing evidence and underscores the need for context-sensitive, transdisciplinary approaches in regions like Egypt, where familial and communicative dynamics may uniquely shape ODD trajectories.

Given prior evidence suggesting associations between early language difficulties, suboptimal maternal psychological and caregiving conditions, and elevated risk for disruptive behavior disorders, we hypothesized that a multidisciplinary intervention simultaneously addressing child language functioning and maternal well-being would be associated with greater improvements in ODD symptoms compared to standard care, with effects maintained at follow-up.

Accordingly, this study aimed to: (1) assess the co-occurrence of pragmatic language deficits and low maternal psychological flexibility in a clinical sample of Egyptian children with ODD, and (2) evaluate the efficacy of an integrated rehabilitation program in improving pragmatic competence, maternal flexibility, and ODD severity, with outcomes measured immediately post-intervention and at a 1.5-month follow-up.

## Methods and patients

### Study design

The current study relied on the case-control prospective follow-up design, which was based on the use of two experimental groups. These groups were subjected to pre- and post-therapy assessment.

A sample of 100 Egyptian children with ODD and their mothers was recruited from the child psychiatry clinic in the period between June 2022 and June 2023. All participants had received a prior clinical diagnosis of ODD from a consultant child psychiatrist based on DSM-5 criteria. Following psychiatric evaluation, children were referred to the Phoniatrics Clinic for a comprehensive language assessment. Pragmatic language abilities were evaluated by a certified phoniatrics consultant using the Egyptian Arabic Pragmatics Language Test (EAPLT) [35], which served as the basis for determining the prevalence of pragmatic language disorder within the cohort.

Of the 100 children with ODD, 60 children exhibiting pragmatic deficits were randomly assigned to two equal groups (*n* = 30/group) using a computer-generated random sequence (1:1 allocation). To ensure allocation concealment and minimize selection bias, the randomization sequence was generated by an independent statistician who was not involved in participant recruitment, assessment, or intervention delivery. Group assignments were placed in sequentially numbered, opaque, sealed envelopes. Each Group consisted of 30 children with ODD and their mothers: Group I received an intervention program that addressed pragmatic language deficits, while their mothers received training sessions for psychological flexibility; Group II received conventional behavioral adjustment sessions. The mothers’ ages ranged from 30 to 45 years, and the children’s ages ranged from 7 to 9 years old. This age range was chosen as it aligns with the most common age at which ODD is presented in the child psychiatry clinic, as stated by Heller [36]. These studies found that 6%–16% of primary school children suffer from ODD. Additionally, pragmatic language development shows a significant increase in complexity after the age of seven [35]. The scarcity of research focusing specifically on girls with oppositional defiant disorder (ODD) is striking. Sanson and Prior [37] highlighted significant gender differences in ODD presentation. Female representation in ODD samples varies considerably depending on the age group studied and the cultural context, with both factors amplifying observed gender disparities. In particular, studies conducted in Western countries during middle childhood often report minimal or no female representation, rendering the current sample exclusively male.

Both groups were matched in age, gender distribution (all male), social standards, total pragmatic language score, severity index of ODD, and total score of the maternal psychological flexibility scale (MPFS). Table 1 displays the baseline data for groups I and II. The socio-economic level was determined using the socio-economic and cultural standard scale developed by Saafan & Khatab et al. [38]. The scale consists of three subscales: economic aspect (13 items), social aspect (5 items), and cultural aspect (7 items). Scores on each aspect were categorized as follows: below average level (1–39), intermediate level (40–79), above average level (80–119), and high level (120–150). Higher scores indicate higher economic, social, and cultural levels. This tool has been validated and proven reliable in determining socio-economic and cultural standards among Egyptian families.

The sampling technique followed an open-label randomized trial to include children based on the following inclusion criteria: (1) Children (boys) who received a diagnosis of ODD according to DSM-5 criteria and were between 7 and 9 years old. Their non-verbal cognitive abilities were ≥ 85, as measured by the Stanford Benet 5th edition [39, 40]; (2) The mothers of the children had low levels of psychological flexibility, as determined by the MPFS.

Exclusion criteria included Children with medical or psychiatric comorbidity, or both, who were excluded from the current study (Children underwent assessment using the mini kids’ tool to quickly review any psychiatric comorbidity) [41], and those who were receiving medical treatment to control ODD or any other medical and psychiatric disorders were also excluded.

All randomized participants completed the intervention and follow-up assessments; therefore, the analytic sample reflects both per-protocol and intention-to-treat principles.

### Intervention

Children in both groups were subjected to four phases.

#### Phase I

Children in groups I and II were assessed through the following:

1. An elementary diagnostic interview, which included clinical history taking. This involved gathering personal data, perinatal history, developmental history, history of present illness, subjective assessment of receptive and expressive language skills and cognitive abilities, schooling, and history of previous rehabilitation, including type, place, and duration.1.An elementary diagnostic interview, which included clinical history taking. This involved gathering personal data, perinatal history, developmental history, history of present illness, subjective assessment of receptive and expressive language skills and cognitive abilities, schooling, and history of previous rehabilitation, including type, place, and duration.
2. Clinical Diagnostic Tools for Children

#### The Egyptian Arabic Pragmatics Language Test (EAPLT) [35]

The EAPLT examined children in various parameters such as non-verbal domain, para-linguistic aspects, understanding inferences, narrative skills, encouraged to ask for information using questions that begin with what, when, where, who, whom, which, whose, why, and how, commonly referred to in linguistics as ‘wh-word questions’ or ‘WH questions’, pragmatics functions, express emotion, and pragmatics factor (identify manners). Each section had its score, and there was also a total score. The test was proven to be reliable, with a Cronbach’s alpha of 0.87. The test determined that children were considered to have a pragmatic language disorder if they scored below the 5th percentile rank for different EAPLTs.

3. Clinical Diagnostic Tools for Mothers

#### The Maternal Psychological Flexibility Scale (MPFS)

This scale measured the level of psychological flexibility among mothers. The scale was developed by translating, combining, and modifying different psychological flexibility scales [42, 43]. The MPFS was developed by adapting core dimensions from internationally validated measures such as the Parental Acceptance and Action Questionnaire (PAAQ) [42] and the Committed Action Questionnaire (CAQ) [44]. The MPFS theoretical framework and item content closely mirror those of cross-culturally validated parental flexibility instruments that consistently predict child behavioral outcomes. The MPFS assesses four dimensions and consists of 28 phrases. These dimensions are positive pressure confrontation, emotion management, problem-solving, and susceptibility to change. This test is a valid and reliable tool for assessing psychological flexibility. Both content and construct validity have been established, with a validity correlation coefficient of 0.83. Reliability has been demonstrated through test-retest reliability, with a P value of 1. The researcher asked the mother to respond to the statement by selecting one of three alternatives (agree, to some extent, or disagree). Each alternative is assigned a score of 3, 2, or 1, respectively. The interpretation of the scale is as follows: higher scores indicate a higher level of psychological flexibility among mothers.

#### ODD scale (Parents form) [18, 45]

This scale serves as a checklist for ODD symptoms and consists of three picture books. For this study, the parents’ picture book was used. The researcher read the displayed phrases, and the mothers were asked to choose the alternative that best described their children’s behaviors. The alternatives are: never happens, sometimes happens (2–3 times/6 h), frequently happens (3–4 times/6 h), very frequently happens (5–6 times/6 h), always happens (occurs all the time). Graded weights were assigned to these responses (0-1-2-3-4) respectively. The higher the score, the more severe the clinical condition is. The scale has been proven to be valid (as demonstrated by agreement validity) and reliable (as demonstrated by test-retest reliability with a *p*-value of 1.00).

##### Phase II

Children in Group I are subjected to the collaborative intervention program (pragmatics language rehabilitation program for children and maternal psychological flexibility training for their mothers).

#### Collaborative intervention program

The intervention program was designed through a collaboration between the professor of psychology and the professor of phoniatrics. The professor of phoniatrics presents the pragmatics rehabilitation program sessions, while the professor of psychology introduces the maternal psychological flexibility training sessions within the intervention program. The program lasts for three consecutive months, with sessions held twice a week. Each session is 2 h long, with 1 h dedicated to pragmatics rehabilitation and the second hour for training the psychological flexibility of the mothers. In total, there are 24 sessions.

#### Pragmatics skills training for children with ODD

Over the course of 12 consecutive weeks, the children participated in twice-weekly sessions, totaling 24 sessions. Each session had a duration of 60 min. The program utilized a variety of strategies, including direct instructions, role-playing, and specific scripts to target various trained skills. The program was individualized for each child based on their pragmatic profile and EAPLT results. We implemented the pragmatics bundle rehabilitation program [46]. This program aimed to improve 13 pragmatic skills, such as understanding gestures and body language, metalinguistic skills, expressing emotions, understanding questions, social interaction, conversational abilities, listening comprehension, abstract adjective comprehension and expression, genderization and pluralization of nouns, figurative language comprehension, and narrative skills. The training of pragmatic skills followed a structured approach, starting with the simplest skills and gradually progressing. We introduced different activities with intermittent cueing until the child demonstrated mastery, which typically took around six weeks. The next six weeks focused on practicing the generalization of these skills to various real-life situations.

#### The maternal psychological flexibility training

The scientific foundation of the program is a Group counselling therapy program that is based on cognitive behavioral theory. The program sessions aim to reduce stress, anxiety, and negative feelings while also increasing psychological flexibility among mothers of children with ODD [47]. Cognitive-behavioral counselling works to correct thinking patterns using techniques that aim to modify the individual’s cognitive structure, leading to positive changes in behaviour [47]. The counselling program consisted of 24 sessions, held twice a week for 12 weeks, with each session lasting 60 min. It adopted the Fundamentals of building a rehabilitation program [48–50]. It explores the general, psychological, cultural, and social foundations and ethics of the psychological counselling process. The aim is to address the four dimensions of psychological flexibility, which include positive confrontation of pressure, emotional management, problem-solving skills training (PSST), and susceptibility to change. Skills in these dimensions are prompted by concepts from cognitive-behavioral theory, such as psycho-education, relaxation skills, problem-solving, activity scheduling, challenging and replacing negative thinking, and social skills.

To target these dimensions, specific strategies are employed, including group lectures, conversations, cognitive reconstruction, self-dialogue, shifting attention towards positive aspects, refutation, stopping negative thinking, positive reinforcement, emotional catharsis, and homework and modelling.

**Problem-solving** is achieved in five steps: identify the problem, brainstorm the solution (without thinking of feasibility), determine the pros and cons of each solution, examine one solution, and finally, discuss whether the option is working [51].

**Group-Lecture technique**: In this technique, participants are provided with information about the nature of ODD and its negative effects on the child and those around them.

**Conversation and discussion technique**: This technique played a crucial role in gaining insight into the psychopathological nature of ODD. It allows individuals to develop a better understanding of the disorder and acquire effective problem-solving strategies.

**The technique of emotional catharsis** is used to release negative and painful feelings and experiences. It is considered one of the most important techniques used for this purpose. These techniques help participating mothers gain insight into their negative thoughts and transform them into positive ones by challenging negative thoughts. Modifying thoughts leads to modifying consciousness and, ultimately, modifying behavior.

**Relaxation techniques** contribute to relieving stress and anxiety, while distraction techniques help mothers escape from their problems by keeping themselves busy with their favourite activities. This not only reduces their burden but also empowers them to regain control over their lives.

**The positive reinforcement technique** increases the participants’ motivation.

### The counseling session scenario

1. At the start of the program, the psychology professor focuses on building strong relationships with the mothers. Then, the program progresses by implementing guidance techniques.
2. The counsellor (psychology professor) shares their opinions without mocking them.
3. Individual differences among the mothers are also taken into account.
4. Group counselling is organized to foster familiarity and encourage interaction among the participants, allowing them to benefit from sharing the same problem.

Children in Group II undergo conventional behavioral adjustment therapy sessions. These sessions are part of a group therapy approach known as Parent Management Training (PMT). The PMT interventions are based on the early work of Gerald Patterson [51], which suggests that ODD is a result of learned behaviors developed through negative interactions between a child and their parent(s). The PMT approaches utilize operant conditioning strategies, such as rewards and punishments, to encourage positive parent/child relationships, increase adaptive behaviors, and decrease disruptive or non-compliant behaviors.

Session attendance was documented for all participants. Participants in Groups I and II attended 98% of scheduled sessions, indicating high adherence to both protocols.

#### Phase III

It entails a post-rehabilitation assessment utilizing the same clinical diagnostic tools conducted in phase I for participants in groups I and II.

#### Phase IV

One and a half months after the program ends, we repeat the clinical and diagnostic tools used in Phase I to assess the maintenance of the outcome.

### Statistical analysis

The data were entered, coded, and processed using the Statistical Package for the Social Sciences. Quantitative data are presented as the mean, standard deviation (SD), and range. The independent samples t-test was used to determine the statistical significance of the difference between two population means in a study involving independent samples. The significance of the results was assessed using a P-value, with values > 0.05 classified as non-significant and values ≤ 0.05 classified as significant. The sample size was calculated to detect a moderate between-group difference (Cohen’s d = 0.5) in pragmatic language skills, maternal psychological flexibility, and ODD symptom severity between children in Group I and those in Group II. Assuming a two-tailed α level of 0.05 and 80% statistical power (β = 0.2), the required sample size was estimated at 80 participants. To account for potential dropouts or incomplete data, this was initially increased to 120. However, due to practical recruitment constraints, specifically, limited referrals meeting strict inclusion criteria (ODD + pragmatic deficit + no comorbidities), the final analyzed sample comprised 60 children (30 per Group). Although this sample size was deemed sufficient for preliminary analyses, it may have limited the study’s ability to detect smaller effect sizes.

## Results

The current study involved a sample of 100 Egyptian children who were diagnosed with ODD according to the DSM-5, along with their mothers. The mothers’ ages ranged from 30 to 45 years, with a mean age of 37.85 years (± 4.80). The children, all of whom were males, were between 7 and 9 years old, with a mean age of 8.15 years (± 0.77). Table 1 provides a baseline description of the children in groups I and II. It compares children in Group I and Group II in terms of their ages, socio-economic status, total pragmatic language skills score, ODD severity index score, and total MPFS score. The significance of the difference between the baseline data of the two experimental groups was determined.

**Table 1:** The baseline description of the children in both groups

Variable	Groups	No.	Mean (SD)	*P* value
Age	Group I	30	8.61 (± 0.69)	1.090
	Group II	30	8.73 (± 0.73)	
Socio-economic standards	Group I	30	101.47 (± 11.85)	**0.290**
	Group II	30	103.15(± 12.49)	
ODD score	Group I	30	91.20 (± 3.82)	0.494
	Group II	30	89.90 (± 4. 91)	
Total pragmatics score	Group I	30	62.00 (± 24.70)	0.623
	Group II	30	63.00 (± 36.76 )	
Total maternal psychological score	Group I	30	29.50 (± 1.96)	0.835
	Group II	30	29.80 (± 2.89)	

Comparing the EAPLT results of children with ODD with their normally referenced peers [35] showed that 60% of children are affected by the following pragmatics deficits:

The younger children displayed poor eye contact in non-verbal aspects. Although they expressed adequate facial expressions, their understanding of these expressions was lacking. They demonstrated adequate use of gestures, but their understanding and use of body distance and posture were affected. Para-linguistic skills exhibited areas of impairment, including the inappropriate use of a loud voice and intonation. Verbal pragmatic skills showed a poor understanding of inferences, especially in certain situations. Their conversational skills were limited, particularly in story-telling from pictures and comprehension of WH questions (especially “where”). Pragmatic factors were severely compromised. The children were unable to express their emotions adequately or identify appropriate manners. Pragmatic functions in the form of “what would you say if” resulted in inappropriate verbal comments. The conversation skills of children with ODD (subjectively assessed) showed that all parameters were affected more than those of typically developing peers. Children with ODD showed significantly poorer attention to their conversational partners. They frequently answered off-target and frequently shifted or changed the topic of the conversation. Most conversational turns were less than three. There were frequent breakdowns in conversation and poor self-repair. The use of clarification requests was unobserved, and the responses to clarification requests were inappropriate. Conversations often ended inappropriately.

### Phase III

#### Pragmatics Language skills assessment

Tables 2 and 3 compare the pragmatic skills of children in groups I and II before and after the rehabilitation program. The data indicate that the program led to improvements in various pragmatic skills. These improvements included better story retelling (*p* = 0.020*), story-telling abilities based on pictures (*p* = 0.040*), enhanced conversational skills (such as an increased number of conversational turns, appropriate use of clarification requests, response to clarification requests, and appropriate conversational termination), as well as an overall higher score in pragmatic skills. However, there were limited changes observed in para-linguistic skills, other conversational skills (like conversational breakdown and using self-repair strategies), and pragmatic factors and functions. On the other hand, Group II, which received the conventional behavioral adjustment program, did not experience the same level of improvement. The mean improvement in total pragmatic score for Group I (Δ = 28.50) was statistically significant, with a 95% CI [12.42, 44.58].

**Table 2:** Pragmatic skills among children in group I Pre- and Post-Intervention

Pragmatics skills	Phase I mean (SD)	Phase III means (SD)	*P* value
Non-verbal aspects	7.50 (± 0.71)	8.00 (± 0.71)	0.200
Paralinguistic aspect	8.00 (± 1.40)	9.00 (± 0.80)	0.080
Understanding inferences:			
Inferences from situations	1.00 (± 1.40)	2.00 (± 0.80)	0.300
Understanding idioms	1.50 (± 2.12)	2.50 (± 0.70)	0.400
Understanding sarcasm	2.00 (± 2.80)	2.00 (± 2.80)	1.000
Narratives			
Story retelling	15.00 (± 14.40)	20.0 (± 7.40)	0.020*
Story telling from pictures	9.50 (± 3.50)	11.00 (± 2.40)	0.040*
What	3.50 (± 0.70)	4.00 (± 0.30)	0.800
Who	3.50	4.00 (± 0.40)	0.900
where	2.00	3.00	0.300
why	2.50 (± 0.70)	3.50 (± 0.40)	0.400
How	1.50 (± 0.70)	2.00 (± 0.60)	0.800
Pragmatics function	13.00 (± 2.80)	14.00 (± 0.80)	0.200
Express emotion	1.50 (± 2.12)	2.00 (± 0.12)	0.800
Identify manners	1.50 (± 2.12)	2.50 (± 0.30)	0.500
Total score for pragmatics	63.00 (± 36.76)	91.50 (± 16.76)	0.001*

*SD* standard deviation

﻿*significant

**Table 3:** Pragmatic skills among children in group II Pre- and Post-Intervention

Skill	Phase I Mean ± SD	Phase III Mean ± SD	Cohen’s d (approx.)	Note
Non-verbal aspects	7.50 ± 0.55	7.50 ± 0.61	0.00	No change
Paralinguistic aspect	8.00 ± 1.10	8.00 ± 1.20	0.09	No significant change
Inferences from situations	1.00 ± 1.10	1.00 ± 1.30	0.16	No significant change
Understanding idioms	1.50 ± 1.60	1.50 ± 2.12	0.00	No significant change
Understanding sarcasm	2.00 ± 2.20	2.00 ± 2.50	0.00	No significant change
Story retelling	15.00 ± 10.95	15.00 ± 13.20	0.12	Not significant
Story telling from pictures	9.50 ± 2.73	9.50 ± 3.20	0.00	Not significant
What	3.50 ± 0.54	3.50 ± 0.70	0.00	No change
Who	3.50	3.50	0.00	No change
Where	2.00	2.00	0.00	No change
Why	2.50 ± 0.54	2.5 ± 0.70	0.00	No change
When	1.50 ± 0.54	-	-	Data missing
How	1.50 ± 0.30	1.50 ± 0.70	0.00	No change
Pragmatics function	1.50 ± 1.60	1.50 ± 2.12	0.00	No change
Express emotion	1.50 ± 1.64	1.50 ± 2.12	0.00	No change
Identify manners	2.50 ± 2.73	2.50 ± 3.53	0.00	No change
Total score	63.00 ± 28.48	63.00 ± 36.76	0.00	No significant change

Bonferroni correction:16 comparisons, adjusted α = 0.003125. After correction, no comparisons were statistically significant

### Maternal psychological flexibility scale

Tables 4 and 5 compare the mean scores of different dimensions of MPFS among mothers in groups I and II before and after rehabilitation. Significant differences were found in all dimensions (*p* = 0.010*). Mothers in Group I showed a large post-intervention increase in total MPFS score (Δ = 52.30, 95% CI [50.10, 54.50]), indicating a robust and reliable effect.

**Table 4:** Comparison of maternal psychological flexibility scale dimensions among mothers in group I Pre- and Post-Intervention

Dimensions	Measurement	Mean (SD)	Z score	*P* value
Positive response to pressure	Phase I	7.30 (± 0.48)	2.83	0.010*
	Phase III	20.20 (± 0.92)		
Emotion management	Phase I	8.80 (± 0.92)	2.82	0.010*
	Phase III	23.00 (± 1.05)		
Problem Solving	Phase I	6.10 (± 0.32)	2.97	0.010*
	Phase III	17.90 (± 0.32)		
Changeability	Phase I	7.30 (± 0.94)	2.97	0.010*
	Phase III	20.60 (± 0.97)		
Total marks	Phase I	29.50 (± 1.96)	2.81	0.010*
	Phase III	81.80 (± 2.09)		

*SD* standard deviation

﻿*significant

**Table 5:** Comparison of maternal psychological flexibility scale dimensions among mothers in group II Pre- and Post-Intervention

Dimension	Phase I Mean ± SD	Phase III Mean ± SD	Z-value	*P*-value	Cohen’s d (approx.)	Note
Positive response to pressure	7.80 ± 1.23	7.60 ± 0.48	-0.52	0.600	0.18	Not significant
Emotion management	8.60 ± 1.07	8.60 ± 1.07	0.00	1.000	0.00	Not significant
Problem Solving	6.50 ± 1.27	6.50 ± 1.27	0.00	1.000	0.00	Not significant
Changeability	7.10 ± 3.16	7.10 ± 0.32	0.00	1.000	0.00	Not significant
Total marks	31.00 ± 4.43	29.80 ± 2.89	-1.45	0.150	0.34	Small-moderate effect

Cohen’s d was calculated using the pooled SD as an approximate measure. Overall, the differences were not statistically significant

### The oppositional defiant scale

Tables 6 and 7 show a statistically significant difference (*p* = 0.010*) in the mean value of the oppositional defiant scale before and after rehabilitation in groups I and II. The reduction in ODD severity among children in Group I was both clinically and statistically significant (Δ = − 60.50, 95% CI [–63.80, − 57.20]).

**Table 6:** Comparison of the oppositional defiant scale total score (Parent Form) Pre- and Post-Intervention among children in group I

Comparison group		No.	Average	standard deviation	z value	Significance level
Group I	before	30	91.20	± 3.82	2.81	0.010*
	after	30	30.70	± 8.23		

**Table 7:** Comparison the oppositional defiant scale total score (Parent Form) Pre- and Post-Intervention among children in group II

Comparison	*N*	Mean ± SD	Z-value	*P*-value	Note
Before intervention	30	89.90 ± 4.65	-	-	Reference
After intervention	30	89.90 ± 4.91	0.00	1.000	No significant change

Cut-off reference: T-score ≥ 70: clinically severe; T-score 60–70: elevated/moderately significant

### The maintenance of the effect of the rehabilitation program after one and a half months

**Fig. 1:**
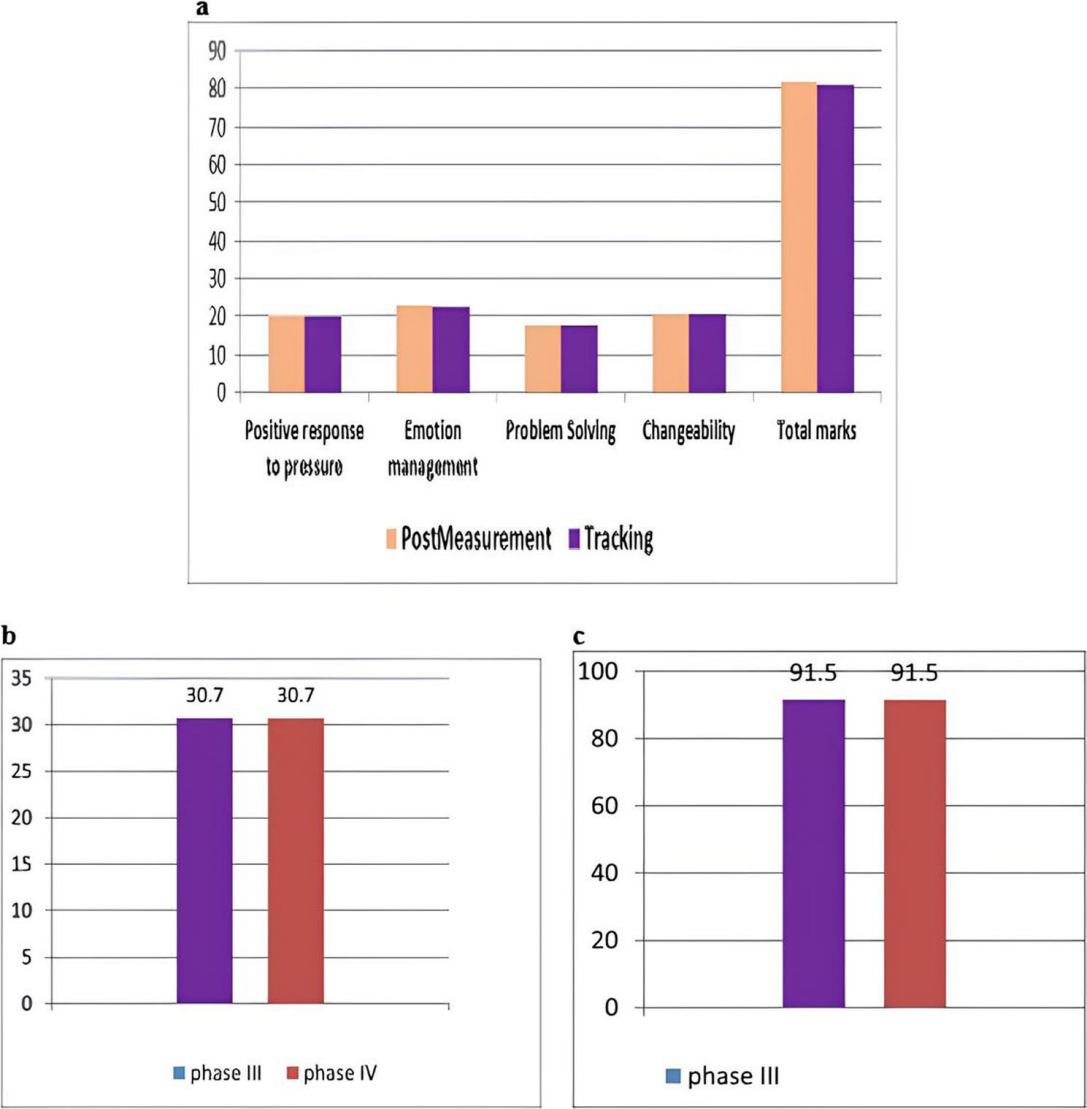
Maintenance of intervention effects at 1.5-month follow-up in Group I (intervention group). **a** Maternal Psychological Flexibility Scale (MPFS) scores at post-intervention (Phase III) and 1.5-month follow-up (Phase IV). Scores for all four dimensions (Positive response to pressure, Emotion management, Problem Solving, Changeability) and Total marks remained stable between assessments (*p* = 1.00 for all comparisons), indicating sustained gains in maternal psychological flexibility. **b** Mean Oppositional Defiant Disorder (ODD) Scale total score (parent form) at post-intervention (Phase III) and 1.5-month follow-up (Phase IV). The significant reduction in ODD symptom severity observed post-intervention was maintained at follow-up (mean score = 30.7 at both time points; *p* = 1.00), demonstrating the durability of clinical improvement. **c** Mean total pragmatic skills score (EAPLT) at post-intervention (Phase III) and 1.5-month follow-up (Phase IV). The significant improvement in children’s pragmatic language abilities achieved through the intervention was sustained at follow-up (mean score = 91.5 at both time points; *p* = 1.00), indicating long-term stability of linguistic gains

There was no statistically significant difference in MPFS total scores among mothers in Group I during phases III and IV (*p* = 1.000; Fig. 1a ). There was no statistically significant difference in oppositional deviant scale scores in Group I during phases III and IV (*p* = 1.000; Fig. 1b). There was no statistically significant difference in EAPLT total scores in Group I during phases III and IV (*p* = 1.000; Fig. 1c).

## Discussion

ODD, one of the most common EBDs, is a challenging disorder that impacts various aspects of children’s lives and their families. Recently, there has been a new theoretical framework analysis on the development mechanisms of these disorders, clinical presentation, and rehabilitation programs that address all these aspects of great variability. Multidisciplinary approaches for managing behavioral disorders, specifically ODD and CD, have been well-established since Eyberg et al. [52]. Advisory Group on Conduct Problems (AGCD) [53] classified intervention programs for EBD into parent and family-based programs (considered the best one by Ward [54], school-teacher and classroom peer-based programs, and multimodal programs. These programs are further categorized based on the level of evidence from review and meta-analysis, ranging from recommended programs to promising programs or programs with a weak evidence base.

This study assessed pragmatic language skills in children with ODD and psychological flexibility among their mothers. Based on these assessments, a multidisciplinary intervention program was developed to address the identified areas of concern. The effectiveness of this program was subsequently evaluated using pre- and post-intervention measures of ODD symptom severity, MPFS, and EAPLT. Pragmatic language serves not only as a communicative tool but also as a scaffold for emotion regulation. Children with ODD often lack the verbal repertoire to label, negotiate, or modulate emotional states in social contexts [55]. When a child cannot articulate frustration or request a turn using socially appropriate language, behavioral escalation (e.g., defiance or aggression) may become the default regulatory strategy. Our intervention directly targeted this link by training children to use pragmatic strategies, such as perspective-taking, emotion labeling, and conversational repair, as alternatives to disruptive behaviors. This aligns with theoretical models positing that language development underpins the internalization of regulatory capacities [56].

The age range of children included in the current work was supported by MacKenzie [57] as the ideal age for responding effectively to behaviour therapy programs. In addition, they should ensure that they do not receive previous training, which could contaminate the results. Also, to introduce a prevention tool for the development of later delinquent behaviour (i.e., antisocial, harmful, and illegal actions committed by young people, primarily under the age of 18, such as theft, vandalism, truancy, substance use, and violence).

Pragmatic language skills are the most commonly affected language parameter among children with behavioral disorders such as ADHD, ODD, and CDs [58]. They are responsible for internalizing behaviors [59]. This deficit may be part of overall linguistic inefficiency, or it may be the most severely compromised linguistic parameter [60]. Pritzker [20] found that despite its importance, there has been a lack of research specifically targeting this aspect. The author explained this lack of research by pointing out the lack of agreement on terms and definitions among disciplines or limited knowledge among professionals in psychology, counselling, education, and social work regarding the commonalities between disciplines. McGuire et al. [61] and Pritzker [20] recommended implementing multiple assessment and management approaches in a single rehabilitation program, also known as interprofessional collaboration.

Therefore, the current study examined pragmatic language skills and found that 60% of children with ODD struggle with pragmatic language disorders. The current work utilized EAPLT as a norm reference tool to compare pragmatic skills among Egyptian children with ODD. This assessment tool is considered an objective, reliable, and valid Egyptian tool for assessing pragmatic skills. The test is provided with a picture book and a recording sheet. Hyter et al. [62] suggested that presenting a pragmatics assessment with visual cues could assist the children being examined. They proposed that visual cues may influence the prevalence of pragmatic disorders. Pritzker [20] found that typically developing elementary school children can complain and express personal opinions, while middle school children should exhibit more sophisticated conversational skills. By high school, assertive communication is mastered. Nippold [63] observed that third-grade children typically develop and can provide listeners with three arguments about a desired object, while seventh-grade children can provide seven.

Helland and Heimann [60] and Benner and colleagues [64] reported that 71% of children with EBD experience clinically significant language deficits. They also confirmed that their sample of children aged 5–13 years with BED showed varying levels of severity in language disorders. Law and colleagues [34] claimed that pragmatic language accounted for 52% of the link between early childhood social disadvantages and later adolescent behavioral disorders. Gilmour and his colleagues [65] found that 78% of children with behavioral disorders received a clinical diagnosis of pragmatic language deficit. This prevalence decreased to 69% and 44% when teachers and parents were asked to assess pragmatic deficits. Pritzker [20] concluded that two-thirds of the clinical sample of children with CD experienced pragmatic language impairment.

In this study, children received a tailored approach to target their pragmatic language skills. Osman [46] developed a comprehensive rehabilitation program for children with primary or secondary pragmatic disorders. The program includes pictures and visual cues to target both non-verbal and verbal pragmatic skills. Walker et al. [66] emphasized the importance of assessing and training pragmatics in a natural context. In this study, children with ODD exhibited compromised pragmatic language skills in several areas. These included: (1) non-verbal pragmatic skills such as body distance, body position, and movement; (2) paralinguistic skills, specifically inappropriate speech loudness; (3) lower ability to make inferences from situations compared to their typical peers; (4) narrative skills, particularly in deducing stories from pictures depicting specific incidents; (5) answering related WH questions; (6) using different verbal speech acts in various situations, such as making requests, giving directives, and making commitments; (7) pragmatic factors, such as describing emotions and identifying bad manners in different situations; and (8) most conversational skills.

Similarly, Hyter et al. [62] found that children with ODD struggled with initiating conversations, understanding requests, and responding to requests for clarification, which hindered their daily conversational interactions. Staikova et al. [67] also discovered a correlation between discourse management and social skills problems in children with behavioral disorders. Additionally, Pritzker [20] noted that children with ODD may experience pragmatic disorders similar to those diagnosed with Autism Spectrum Disorder (ASD). Salmon et al. [68] supported the significant role of emotional expression in behavioral development. O’Kearney & Dadds [69] suggested that the clinical presentation of internalizing and externalizing behaviors negatively impacts “emotional language.” Among their sample, affected adolescents were unable to express emotions in situation-specific ways. In response to these challenges, several studies have developed pragmatics-based language intervention programs. Fleming et al. [70], Laugeson et al. [71], and Obsuth et al. [72] focused on improving conversational skills, such as initiating and ending conversations appropriately. In this study, improvement in narrative and conversational skills was observed after the post-rehabilitation assessment. However, there was limited improvement in pragmatic functions and factors, which may require a longer duration of rehabilitation.

The intervention plan proposed by Hyter et al. [62] closely resembled the program introduced in this study. Their intervention program targeted sequencing events, expressing opinions about inappropriate behaviors (judgment), describing objects, and negotiating desired outcomes. In their results, five out of six participants improved their scores in object description, and four out of six improved their scores in step-by-step directions. Arguing about desired outcomes was a challenging skill. They utilized strategies such as direct structuring, specific scripts for different situations, and role-play modelling.

Hyter et al. [62] also followed the same order of difficulty for pragmatic skills as this study: describing an object (the least difficult), providing sequenced directions (medium difficulty), and expressing opinions about inappropriate behaviors (highly difficult). Negotiating reasons for certain desired objects was considered the most sophisticated pragmatic skill. They claimed that practicing pragmatic skills in contexts without visual cues may require different skills compared to those with visual cues, as the speaker should be able to assume the listener’s presuppositional knowledge.

In contrast to the findings of this study, Obsuth et al. [72] and Hayman [73] disagreed. Obsuth et al. [72] exposed secondary school participants, with an average age of 14.5 years and diagnosed with behavioral disorders, to an intervention program aimed at improving conversational skills. The program focused on teaching participants how to ask for clarification and interrupt the speaker appropriately. The outcomes were assessed using behavioral disciplinary measures, and no significant difference was found between the intervention and control groups. It was suggested that the limited focus on conversational skills and the older age of the participants may have contributed to the contradictory findings compared to this study. Hayman [73] reported non-significant changes in social skills following an intervention program that included pragmatic skills rehabilitation.

Morshed et al. [74] examined the effectiveness of play therapy, either individually introduced or in groups, for controlling symptoms of a group of children with ODD aged 6–10 years. Their results were based on parental and teachers’ ratings of ODD symptoms. There was a significant decrease in ODD symptoms in the experimental Group when compared to the control group. This effect was consistent for 2 consecutive months. Group therapy influenced the parental rating (especially the mothers) of ODD symptoms more significantly than individual therapy. They commented on the role of group play therapy in promoting social interaction and communication skills. They highlighted the mechanism of individual play therapy in improving emotional discharge, reducing painful effects, and correcting emotional thrill, while the group therapy targeted others’ acceptance, friendship relationships, and social interaction skills. The first modality is believed to be an emotional treatment, while the second is a social skills enhancer.

This intervention program focuses on changing parent behaviors, child functioning, and parent-child interactions [75]. According to Kazdin [76], prosocial behavior increases due to changes in parental strategies, behaviors, and procedures. Daks et al. [77] argue that psychological flexibility mediates parental pressures and positive psychological outcomes. Several prior studies have explored the positive connection between positive parenting practices and psychological flexibility [78–81]. Brassell et al. [78] and Eisenberge et al. [82] found that higher levels of maternal psychological flexibility were associated with decreased internalizing and externalizing behaviors. This effect was mediated by children’s emotional regulation. Ren et al. [29] explored the relevant influencing factors that mediate behavioral disorders among preschool Chinese children. Their work illustrated the role that parents’ psychological flexibility plays in mediating emotional regulation among preschool children.

In the current work, the maternal psychological flexibility rehabilitation program is developed by integrating different psychological flexibility skills [42, 43, 83]. The program covers four dimensions. The first is positive pressure confrontation, in which mothers are trained to use positive methods to cope with psychological pressures in their daily lives. The second dimension is emotional management. The training in this dimension enhances the maternal ability to control emotions, understand their own and others’ feelings, and share their feelings with others. The third dimension is related to problem-solving strategies. Mothers are taught how to implement step-by-step strategies to overcome difficulties, including finding positive solutions and applying the easiest ones to solve problems. The fourth dimension is related to enhancing susceptibility to change. In this dimension, mothers learn to accept the principle of change and act flexibly with events if necessary. Spruijt et al. [83] supported the idea that excellent parenting strategies are essential to meet children’s needs and positively impact emotional regulation among children. Psychological flexibility improves parenting practices and attitudes.

Ren et al. [29] determined that parents’ psychological flexibility includes cognitive diffusion, committed action, and acceptance. Cognitive diffusion refers to parents’ ability to consciously separate their negative emotions from their parenting behavior. Committed action means that parents can fully respect their children’s wishes and allow them to maintain appropriate independence. Acceptance means that parents accept the painful emotions and thoughts that arise during parenting [42]. They hypothesized that parental psychological flexibility acts as a mediator of their children’s emotional regulation, which could promote positive social adaptation. The sustained improvement in ODD symptoms likely reflects a reciprocal reinforcement loop between child communication gains and maternal psychological flexibility. As mothers learned to respond with greater adaptability and less reactivity (e.g., through cognitive defusion and committed action), they created a calmer interpersonal climate that supported the child’s practice and generalization of pragmatic skills. Conversely, as children’s ability to express needs and interpret social cues improved, daily interactions became less conflictual, thereby reducing maternal stress and further enhancing parental flexibility. This bidirectional dynamic, consistent with transactional models of development [29], may explain why gains were maintained at the 1.5-month follow-up, even without booster sessions.

In this study, the MPFS increased following training in these skills. Daks et al. [77] claimed that psychological flexibility improves parent-child interaction. Following the implementation of the current intervention program among participants in Group I, the Oppositional Defiant Scale-Parent form score markedly declined compared to pre-intervention levels. This finding is supported by Williams et al. [84] and Brown et al. [85], who suggest that higher levels of psychological flexibility among parents are associated with a reduction in family conflicts and fewer psychological disorders in children, particularly those related to anger, aggression, and negative mood.

MacKenzie [57] discussed the parent-based approach for treatment of ODD under the umbrella of Behavioural Parent Training (BPT). The mechanism of action of parent training is thought to be a preventive technique that targets increasing positive child behaviour, responding to their children’s disruptive behaviour. In addition to this, it reduced classroom behaviours. They emphasized that adding a centred approach and/ or teacher-oriented approach may increase the strength of the outcome of any BPT. Kazdin and Whitley [86] suggested that BPT, which included problem-solving techniques in children aged 6–14 years, mediated a positive outcome for child behaviour. Breitenstein et al. [87]also used naturalistic videos of African American parents effectively using the techniques, instead of verbal descriptions of techniques or videos with professional actors. The author claimed that this technique is more accepted by parents of children with ODD aged 2–5 years old. Snell-Johns et al. [88] supported the use of home-based and self-directed, especially for unfavourable cultural and social backgrounds.

Consistent with our study, Fleming et al. [70] found that the reduction in depressive symptoms among their participants could still be observed up to four weeks after the intervention program ended. Hayman [73] followed the outcome of their intervention program and observed a decline in verbal and physical aggression among the participants. However, our findings were opposed by Obsuth et al. [72], who found no statistically significant differences in behavioral measures after five months following the intervention. While the intervention effects were maintained at the 1.5-month follow-up, a promising indicator of short-term stability, the brevity of this interval limits our ability to infer sustained or long-term outcomes. This follow-up duration was selected pragmatically to balance the need for initial maintenance data against the high risk of attrition in longitudinal behavioral studies within our clinical setting. Future research should incorporate extended follow-up assessments at 3 and 6 months post-intervention to more rigorously evaluate the durability of gains in pragmatic skills, maternal psychological flexibility, and ODD symptom reduction.

Hyter et al. [62] was the only study that objectively assessed pragmatic language skills. Although the positive outcomes of their intervention program did not reach statistical significance, some pragmatic skills were acquired. The researchers attribute this to a small sample size, lack of control, and a short intervention period.

The current work is considered one of the few works that addressed and integrated two rehabilitation programs for children with ODD and objectively assessed their outcomes from different aspects. There is limited collaboration between different disciplines, which hinders the provision of an adequate rehabilitation program for children with ODD [89]. Chong et al. [90] and Strunk et al. [91] support the universality of this problem and suggest that regulatory roles, time constraints, and fears of conflicts between different disciplines may be the underlying reasons.

### Limitation

Some areas need inspection in future work. First, the absence of females in the current work limits information about the nature of the disorder in different genders. Second, the limited inclusion of the age range between 7 and 9 years only significantly restricts the generalizability of our findings. The absence of a no-treatment or waitlist control group. Both study arms received active interventions, either the Collaborative Intervention Program or the conventional behavioral adjustment program, which limits our ability to rule out the influence of non-specific factors (e.g., participant expectations, repeated testing, or general therapeutic contact) on the observed outcomes. Future studies would benefit from including a third, untreated control group to more rigorously isolate the specific effects of the multidisciplinary program. Future work should study the profile of ODD among younger and older age ranges. Additionally, reliance on maternal self-report for both psychological flexibility and child ODD symptoms may introduce shared-method variance or perceptual bias, potentially inflating observed associations. Another limitation is the lack of blinding of outcome assessors. The same clinicians who delivered the intervention also conducted all assessments, which may have introduced bias in the evaluation of outcomes. Future studies should employ independent, blinded assessors to enhance objectivity. While the ODD scale provided a continuous measure of symptom severity, no universally validated cut-off score was available in the version used. Therefore, the interpretation of ODD severity in this study was based on relative comparisons between groups rather than categorical thresholds. This methodological limitation should be considered when generalizing the findings.

## Conclusion

This study provides promising preliminary evidence that a collaborative intervention targeting pragmatic language skills in children with ODD and psychological flexibility in their mothers may be associated with meaningful reductions in ODD symptom severity. While the results suggest potential clinical utility, they should be interpreted with caution due to methodological limitations. Larger, more rigorously controlled trials are needed to confirm these findings and establish the intervention’s efficacy and durability over time.

### Recommendation

Based on the findings of this study, the following recommendations are proposed. First, there is a clear need to develop a comprehensive, developmentally integrated management protocol that addresses the full spectrum of developmental domains, including cognitive, linguistic, socio-emotional, and behavioral functioning, in children with EBD. Second, recognizing the bidirectional relationship between language and behavior, children diagnosed with speech and language disorders, particularly pragmatic language impairments, often exhibit co-occurring challenges across multiple developmental areas, necessitating systematic, multidisciplinary assessment and coordinated intervention. Third, future research should prioritize large-scale, school-based studies involving children with EBD across diverse age groups to establish robust epidemiological data on the prevalence and developmental trajectory of pragmatic language difficulties within this population, thereby informing early identification and targeted support strategies.

## Data Availability

The research data are available upon reasonable request from the corresponding author.

## Abbreviations

ADHD: Attention Deficit Hyperactivity Disorder
AGCD: Advisory Group on Conduct Problems
ASD: Autism Spectrum Disorder
CBT: Cognitive behavioral therapy
CD: conduct disorders
DSM-5: The Diagnostic and Statistical Manual of Mental Disorders, Fifth Edition
EAPLT: Egyptian Arabic Pragmatics Language test
EBD: Emotional behavioral disorders
EBD: Emotional Behavioral Disorders
MPFS: The Maternal Psychological Flexibility Scale
ODD: Oppositional Defiant Disorder
SD: Standard Deviation
